# A Case of Quadruplets

**Published:** 1887-07

**Authors:** S. T. Lowry

**Affiliations:** San Antonio, Texas


### A CASE ON QUADRUPLETS.

*By S. T. Lowry, M.D., San Antonio, Texas.*

For Daniel's Texas Medical Journal.

IN COLLINS table of over 129,000 women delivered in the Dublin Lying-in-Hospital there were over 2000 cases of twins, 29 triplets, and only one of quadruplets, while in the Royal Maternity Charity out of 50,000 successive cases of labor, there was not a single case of quadruplets. While tables showing preternatural fecundity may vary in different nations, and at different times, still, we may ordinarily expect twins in every 75 or 80 births, triplets in every 5,000 births, while quadruplets are so rare as to defy any kind of accurate calculation. The rarity of such cases is my only apology for briefly mentioning a case of quadruplets that came under my observation on the night of the 28th ultimo. Mrs. F., a German woman about 35 years old, who had borne several children by a former husband, and who had been married to her second husband about one year, was taken in premature labor at about six months of utero-gestation, on 28th of June, and was delivered of four well developed and well nourished female children. They were all of uniform size, measuring about twelve inches in length, and weighing about two and one-half pounds each, They all showed some signs of life, and one lived some minutes, and cried faintly. Each child was contained in separate membranes, with separate cord and placenta, but the placentæ had encroached, in their growth, upon the margins of each other, and were attached at their edges. The placentæ were removed with some difficulty from their attachment, and there was considerable hemorrhage; but the mother made a rapid and good recovery.

